# Anthracyclines and trastuzumab associated cardiotoxicity: is the gut microbiota a friend or foe? – a mini-review

**DOI:** 10.3389/frmbi.2023.1217820

**Published:** 2023-09-08

**Authors:** J. Guilherme Gonçalves-Nobre, Inês Gaspar, Diogo Alpuim Costa

**Affiliations:** ^1^ Hospital Garcia de Orta E. P.E, Lisbon, Portugal; ^2^ Instituto de Saúde Ambiental e Saúde Pública (ISAMB), Lisbon, Portugal; ^3^ Instituto de Medicina Preventiva e Saúde Pública (IMP&SP), Lisbon, Portugal; ^4^ PTSurg – Portuguese Surgical Research Collaborative, Lisbon, Portugal; ^5^ Haematology and Oncology Department, CUF Oncologia, Lisbon, Portugal; ^6^ Medical Oncology Department, Hospital de Cascais Dr. José de Almeida, Alcabideche, Portugal; ^7^ Faculdade de Ciências Médicas, NOVA Medical School, Lisbon, Portugal

**Keywords:** gut microbiome, gut microbiota, chemotherapy, anthracyclines, doxorubicin, trastuzumab, cardiotoxicity

## Abstract

Breast cancer (BC) is one of the most prevalent cancers worldwide. Fortunately, BC treatment has taken a huge turn in the last few years. Despite these advances, one of the main issues related to systemic treatment remains the management of its side effects, including cardiotoxicity. In this regard, we highlight the irreversible dose-dependent cardiotoxicity of anthracyclines related to oxidative stress and the reversible cardiotoxicity with trastuzumab, whose mechanism is still poorly understood. Moreover, the combination of anthracyclines and trastuzumab further exacerbate the myocardial damage. More recently, altered gut microbiota composition has been linked to the long-term effects of cancer therapy, including the potential connection between treatment-related microbial changes and cardiotoxicity. *Bacteroides* spp., *Coriobacteriaceae*_UGC-002, and *Dubosiella* have already been reported as bacterial species with deleterious effects on the myocardium, mainly due to the promotion of inflammation. On the other hand, *Alloprevotella*, *Rickenellaceae*_RC9, *Raoultella planticola*, *Klebsiella pneumoniae*, and *Escherichia coli* BW25113 can induce cardioprotection, predominantly by increasing anti-inflammatory cytokines, promoting intestinal barrier integrity and early metabolization of doxorubicin. Herein, we explore the role of gut microbiota in the development of cardiotoxicity, as well as future perspectives to decrease the risk of cardiotoxicity associated with BC treatment.

## 1 Introduction

Breast cancer (BC) is the most common cancer among women and the second most common worldwide. This type of cancer is a multifactorial disease, and several factors, such as race, ethnicity, and demographic characteristics, contribute to its incidence. It is expected that the BC mortality rate will increase by 2030, particularly in developing countries, despite all screening measures and the evolution of the therapeutic armamentarium (Adeoye and Adeoye, 2023).

Molecular BC subtypes determine prognosis and indication of specific systemic therapy, including endocrine therapy for hormone receptor-positive tumors (with some patients also requiring chemotherapy), trastuzumab-based therapy plus chemotherapy for human epidermal growth factor receptor 2 (HER2) tumors, and chemotherapy alone or combined with immunotherapy for triple-negative BC (Waks and Winer, 2019). Chemotherapy is still an essential treatment for many patients with stage I-III BC, despite the potential short- and long-term side effects (Waks and Winer, 2019).

Among the drugs most used in the treatment of BC, anthracyclines and trastuzumab stand out. Nevertheless, the toxicity of these drugs should not be underestimated. A well-known side effect is a cumulative cardiotoxicity, the main reason for dose-limited administration (Carvalho et al., 2009; Barish et al., 2019).

As such, efforts should be made to find more effective measures to overcome these drugs’ toxicity while maintaining or enhancing their therapeutic efficacy. Gut microbiota dysbiosis has recently come to light as a significant player that may impact BC development, therapy, and prognosis through a various molecular mechanism. Therefore, gut dysbiosis can potentially affect the responses and toxicity profile of antineoplastic agents (Alpuim Costa et al., 2021).

Hence, the aim of this review is the exploration and clarification of the links between the influence of gut microbiota and cardiotoxicity induced by antineoplastic drugs, namely anthracyclines and trastuzumab.

## 2 Anthracyclines associated cardiotoxicity – mechanism of action

Doxorubicin (DOX) belongs to non-selective class I anthracycline family. Its clinical use is known for cumulative and irreversible cardiotoxicity, which leads to aberrant arrhythmias, ventricular dysfunction, and congestive heart failure, even years after chemotherapy cessation. Among all theories that have been proposed, the more accepted and defined are mitochondrial dysfunction, DNA damage, defects in iron metabolism, and higher levels of glucose consumption (McGowan et al., 2017; Henriksen, 2018).

**Figure 1:**
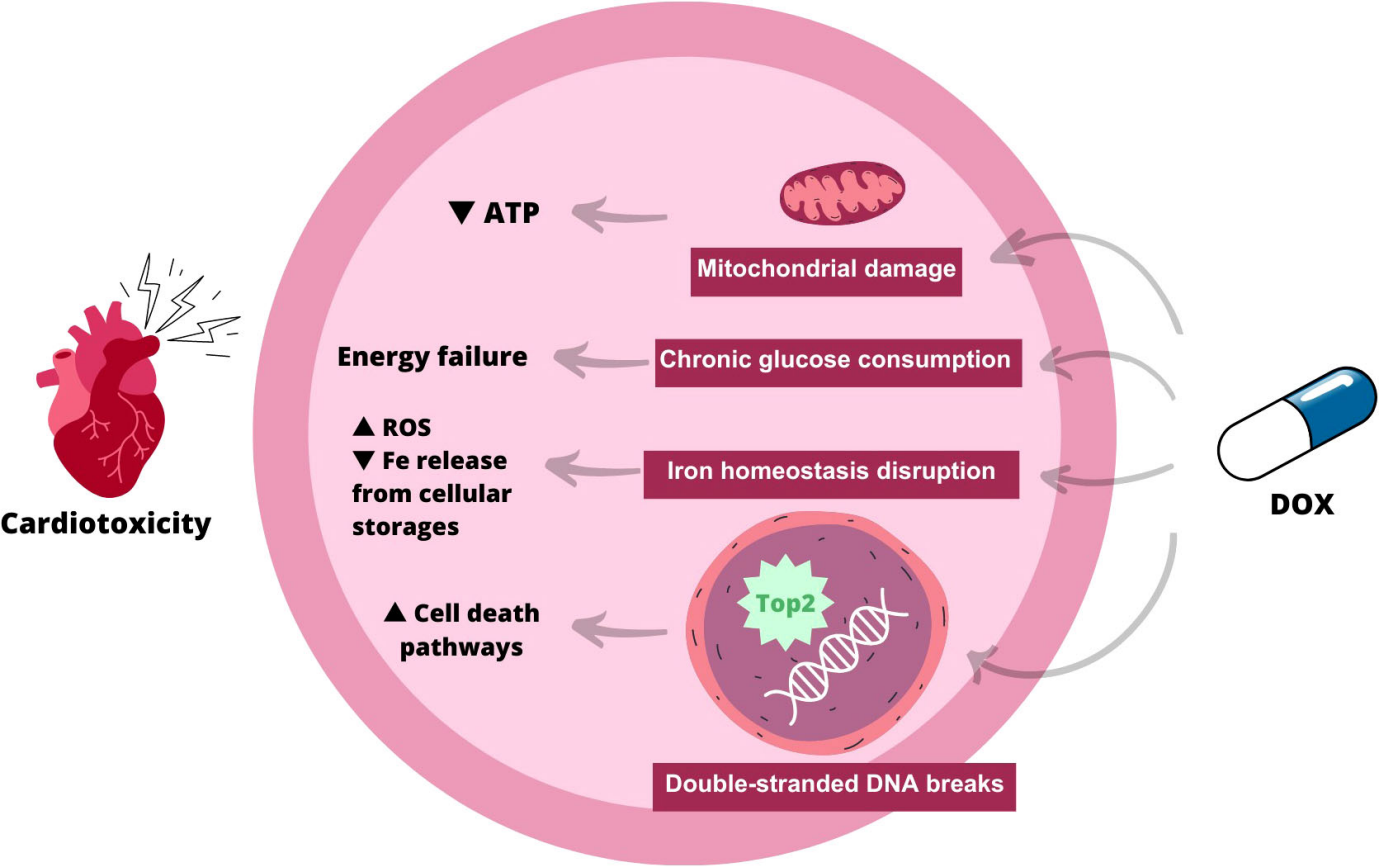
Scheme explaining the mechanism of action of doxorubicin-induced cardiotoxicity. As indicated, when doxorubicin is administered, there are several possible pathways: 1) Mitochondrial damage, leading to a decrease in ATP within myocardial cells; 2) Chronic glucose consumption, which contributes to energy failure; 3) Iron homeostasis disruption, which increases reactive species of oxygen and decreases iron from cellular storages; 4) Induction of double-stranded DNA breaks, which leads to increased apoptosis. ATP, Adenosine Tri-phosphate; ROS, Reactive Octive Species.

The hallmarks of DOX-induced cardiotoxic effects have been shown to include mitochondrial damage and accumulation of dysfunctional mitochondria (**Figure 1**). By binding to cardiolipin, DOX accumulates in the inner mitochondrial membrane and decouples the respiratory chain complexes, which reduces ATP synthesis. Thus, DOX cardiotoxicity directly contributes to ATP deficiency by altering mitochondrial energy metabolism and bioenergetics (Henriksen, 2018).

Regarding DNA damage, the therapeutic effect of anthracyclines against cancer cells is mediated by the inhibition of topoisomerase (Top) 2α, increasing DNA breaks, and preventing DNA and RNA synthesis. In cardiomyocytes, Top 2β is also inhibited, causing double-stranded DNA breaks. The Top 2 inhibition in both cell types causes accumulation of double-stranded DNA breaks and mitochondrial dysfunction, leading to activation of cell death pathways and accumulation of reactive oxygen species (ROS) (McGowan et al., 2017; Henriksen, 2018).

Impairment of mechanisms involved in cellular iron homeostasis can occur through different mechanisms:

### 2.1 Formation of ROS

Within the cell, DOX is reduced to a cytotoxic semiquinone radical which is rapidly converted back to the original molecule using O_2_ as an electron acceptor, leading to the formation of superoxide formation that is detoxified in H_2_O_2_. Secondly, the labile iron pool (LIP) - cellular pool of cheatable and redox-active iron – reacts strongly with H_2_O_2_, generating ROS via Fenton´s reaction. Furthermore, LIP can directly interact with DOX, creating DOX-Fe complexes that drive ROS production.

### 2.2 Disruption of iron localization in the cell

DOX modulates mRNA maturation of transferrin receptor and ferritin through irreversible inactivation of the RNA-binding activity of iron regulatory proteins 1 and 2. This mechanism increases iron/ferritin binding in the cytosol and reduces its release from cells storages, including mitochondria. Iron accumulation in mitochondria has been linked to ferroptosis, which is believed to play a role in DOX-induced cardiomyopathy.

Fatty acids are the primary fuel for catabolic reactions in cardiac metabolism under normal circumstances, while glycolysis is used in response to pathological events. Doxorubicin enhances serum triglyceride and blood glucose levels and at the same time triggers massive cardiac glucose uptake due to AMPK inhibition and concomitant expression of GLUT1 (normally absent in the adult heart). Chronic glucose consumption eventually becomes maladaptive and results in energy failure. Additionally, a clinical investigation found that patients with comorbidities conditions such as diabetes, dyslipidemia, and obesity are at increased risk of DOX-induced cardiotoxicity (Russo et al., 2021).

Although doxorubicin is the most studied anthracycline, in terms of chemotherapy-induced cardiotoxicity, there are a few studies exploring the same side effects in other anthracyclines, such as epirubicin, as well as anthracenedione (i.e. anthracycline analogue), namely mitoxantrone.

Apparently, even in the second generation of anthracyclines, namely with idarubicin or epirubicin, cumulative cardiotoxicity remains a relevant side effect (Minotti et al., 2004; Morelli et al., 2022). Furthermore, it was noted that also anthracyclines derivatives, such as Mitoxantrone, can induce myocardium lesions, leading to cardiac failure, however with a significant safer profile than doxorubicin (Saletan, 1987; Damiani et al., 2016; Morelli et al., 2022).

## 3 Trastuzumab associated cardiotoxicity – mechanism of action

Trastuzumab is a humanized monoclonal antibody that blocks the action of HER2. Its precise mode of action against cardiomyocytes is still uncertain. Nevertheless, cardiomyocyte growth and proliferation are significantly influenced by HER2 and the ErbB family of tyrosine kinase receptors. Thus, blocking of downstream intracellular signaling by trastuzumab may affect cellular metabolism, leading to sarcomere disruption and impaired cell proliferation. In addition, DOX and trastuzumab can have a synergic effect, as the former initiates an oxidative damage process and the latter blocks HER2 downstream signaling, essential to cellular repair (Barish et al., 2019).

The main mechanism of action of trastuzumab stands for binding to HER2 receptors, leading to the blockade of their dimerization and, subsequently, their downstream signaling. However, it was recently unveiled a new side mechanism that involves the immune system. Exactly after the connection of antibody-receptor, it occurs an interaction between the Fc receptor of anti-Her2 antibody and FC receptors of immune cells, such as neutrophils, NK cells, γδ T cells and macrophages. As a consequence, it activates the immune system, conducting to an enhanced presence of tumor antigens in the tumor microenvironment, which leads to increased cytotoxicity, with the aim to augment the efficacy of antigen-presenting cells (APC). Moreover, NK cells in one way, improve the cytotoxicity of CD8+ T cells, through dendritic cells priming, as well as induce a Th1 phenotype and, in another way, NK cells promote a pro-tumoral cytokine production, recruiting even more T and myeloid cells (Bianchini and Gianni, 2014).

## 4 Effect of gut microbiota on cancer treatment-associated cardiotoxicity

### 4.1 Anthracyclines - Doxorubicin

Although the mechanism of DOX-induced cardiotoxicity is becoming clearer, there are still some gaps that might be fulfilled by microbiota research. Recently, a considerable number of studies have shifted the focus from chemotherapy-induced cardiotoxicity to a possible cause related to gut dysbiosis. Indeed, it has been observed that after DOX treatment, there are three main impacts on the intestinal microbiota (**Table 1**): 1) Direct lesion of the intestinal mucosa; 2) Structural/composition changes; 3) Metabolomic modifications.

**Table 1:** Microbiota effects in doxorubicin-induced cardiotoxicity.

Bacteria/Metabolite	Mechanism	Type of study	Ref
1. Direct lesion on intestinal barrier			
Cluster of lymphocytes, gut ulcers and loss of goblet cells		Animal	(Chelakkot et al., 2018; An et al., 2021)
Increase concentration of endotoxins		Animal	(Chelakkot et al., 2018; An et al., 2021)
Decrease of tight-junction proteins, such as ZO-1		Animal	(Chelakkot et al., 2018; Huang et al., 2022)
2. Microbiota Composition Changes			
***Bacteroides* spp.**	Modification of gut microbiota network	Animal	(Rocha and Smith, 2013; Huang et al., 2022)
***Actinobacteria - Coriobacteriaceae***	Modification of colonic macrophages into M1-like pro-inflammatory macrophages with increased levels of TNF-α and IL-1β	Animal	(Liu et al., 2020; Lin et al., 2021)
***Coriobacteriaceae_UGC-002***	Increase myocardial enzymes, which means increased myocardial damage	Animal	(Liu et al., 2020)
***Dubosiella***	Increase myocardial enzymes	Animal	(Guo et al., 2018; Liu et al., 2020)
***Alloprevotella***	Increase intestinal barrier integrity, prevent pathogen proliferation and substrate for colonocytes	Animal	(Dubin et al., 2016; Huang et al., 2022)
***Rikenellaceae_RC9***	Same as previous effects. Attenuate colitis through CTLA-4 disruption by increasing the regulatory T-cells differentiation	Human	(Dubin et al., 2016; Yan et al., 2018)
***Enterobacteriaceae – Raoultella planticola***	Metabolize doxorubicin into 7- deoxydoxorubicinol and 7-deoxydoxorubicinolone, which are inactive metabolites	Human	(Alpuim Costa et al., 2021; Mamic et al., 2021)
***Klebsiella pneumoniae & E. coli* BW25113**	Inactivate doxorubicin through molybdopterin-dependent enzymes	Human	(Alpuim Costa et al., 2021; Mamic et al., 2021)
3. Metabolome modifications			
**Secondary bile acids**	Increase triglyceride gathering and inflammation, leading to myocardial apoptosis and fibrosis	Animal	(Romano et al., 2015; Liu et al., 2020)
**TMAO**	Increase inflammation through induction of NLRP3 inflammasome, leading to cardiac fibrosis. Also, induces differentiation of monocytes into macrophages and foam cells, contributing to atherosclerosis development	Animal/ Human	(Brown and Hazen, 2018; Li et al., 2019; Liu et al., 2020; Xiong et al., 2022)
**SCFA**	Anti-inflammatory effects. Decrease ROS.	Animal	(Carvalho et al., 2009; Liu et al., 2020)

Green color corresponds to beneficial effects and red is associated with deleterious effects. It is divided in 3 parts: 1. Direct lesion of the intestinal barrier; 2. Microbiota composition changes; 3. Metabolomics modifications. ROS, Reactive oxygen species; TMAO, Trimethylamine N-oxide; SCFA, Short-chain Fatty Acids; ; NLRP3, NOD-, LRR- and pyrin domain -containing protein 3; CTLA-4, Cytotoxic T-Lymphocyte- associated protein 4; ZO-1, Zonula occludens 1; IL, Interleukin; TNF, Tumour Necrosis Factor.

#### 4.1.1 Direct lesion of the intestinal mucosa

It has been reported that patients undergoing chemotherapy frequently develop acute intestinal mucositis (Bianchini and Gianni, 2014), and so An L. et al. explored DOX-induced damage to the intestinal barrier. As a result, after DOX administration, via intraperitoneal injection, in a dosage of 5 mg/Kg, there was infiltration of lymphocytes clusters, gut ulcers, and loss of goblet cells, which gave rise to endotoxemia (Szeto et al., 2018; An et al., 2021).

Furthermore, the concentrations of tight-junction proteins, like claudins, occludins and, particularly, zonulin (ZO-1), whose function is to stabilize the intestinal membrane, were reduced in the DOX group. When a fecal microbiota transplant (FMT) was performed, ZO-1 levels were restored and the concentration of endotoxins was decreased, which means that this chemotherapy induces damage to the the intestinal wall and, one of the possible approaches to reduce this side effect, could be the FMT (Chelakkot et al., 2018; An et al., 2021).

#### 4.1.2 Microbiota structural/composition changes

First, a significant decrease in alpha diversity was observed (Junpaparp et al., 2013). Moreover, it was possible to correlate some bacterial modifications with cardiotoxicity outcomes.

Starting with bacteria with deleterious effects, in terms of cardiotoxicity, *Bacteroides* spp. was identified as a relevant harmful microorganism that influences the entire gut microbiota network. This bacterium is a gram-negative and obligate anaerobic, with the characteristic of being an opportunistic pathogen in infections (Rocha and Smith, 2013; Huang et al., 2022).

Furthermore, the phylum *Actinobacteriota* was also increased in mice under DOX treatment (by intraperitoneal injection, with a cumulative dosage of 20 mg/Kg), suggesting a negative impact on cardiovascular disease, in Liu et al. study, it was even observed that a family from the abovementioned phylum, named *Coriobacteriaceae*, was actually increased and contributed to modifying colonic macrophages into a pro-inflammatory M1 macrophag, which amplified the concentrations of TNF-α and IL-1β, pro-inflammatory cytokines that promote cardiotoxicity (Liu et al., 2020; Huang et al., 2022). Additionally, one genus of this family, *Coriobacteriaceae*_UCG-002, had a particularly higher concentration compared to other genes and was associated with serum myocardial enzymes, pointing to a potential increase in DOX-induced cardiac lesion (Huang et al., 2022). Alongside with this cardiac condition, another genus was correlated with increased myocardial enzymes, namely *Dubosiella* (Huang et al., 2022). In fact, *Dubosiella* was reduced in a study that revealed beneficial outcomes from yellow wine compounds, demonstrating that their absence resulted in enhanced effects. In this study, mice were submitted to a DOX treatment, via intravenous tail injections, with a dosage of 4 mg/Kg, one time per week, for 4 weeks straight (Lin et al., 2021).

In another perspective, the intestinal microbiota can also protect against DOX-induced cardiotoxicity, through different mechanisms. One of them comprises the strengthening of the intestinal barrier by increasing the levels of *Alloprevotella* and *Rikenellaceae*_RC9 and decreasing of *Prevotellaceae*_UCG-001. All referred bacteria are producers of short chain fatty acids (SCFA), namely acetate, propionate, butyrate and valeric acids, using mucin as a substrate. These SCFAs are important because they can prevent pathogenic proliferation, uphold the intestinal barrier, and provide food for colonocytes (Guo et al., 2018; An et al., 2021). *Rikenellaceae*_RC9 also has the ability to decrease colitis symptoms through anti-inflammatory mechanisms and CTLA-4 disruption by increasing the differentiation of regulatory T-cell (Dubin et al., 2016; An et al., 2021).

On the other hand, the other beneficial mechanism is the early metabolization of DOX. *Raoultella planticola*, under specific anaerobic conditions, can inactivate DOX through reductive deglycosylation, originating 7-deoxydoxorubicinol and 7-deoxydoxorubicinolone, inactive metabolites and thus reducing the bioavailability and toxicity of DOX. Furthermore, some bacteria, such as *Klebsiella pneumoniae* and *Escherichia coli* BW25113, can metabolize DOX into inactive metabolites, with a different mechanism using molybdopterin-dependent enzymes (Yan et al., 2018; Alpuim Costa et al., 2021).

#### 4.1.3 Metabolomic modifications

Doxorubicin has been shown to modify metabolic pathways such as the biosynthesis of glycans, amino acid, lipids and other complimentary metabolites. Moreover, even the microbial metabolites are altered after administration of this chemotherapeutic agent, such as SCFA, trimethylamine N-oxide (TMAO) and other aminoacidic chemicals. and therefore, may contribute to DOX-induced cardiotoxicity (Mamic et al., 2021).

Some bacterial metabolites have beneficial effects on the mechanism of cardiotoxicity, like SCFA, but, on the other hand, some contribute and even exacerbate the deleterious consequences of DOX use, such as TMAO and secondary bile acids.

Secondary bile acids consist of primary bile acids converted by bacterial enzymes, such as 7α dihydroxylase, which can be found in the small intestine, and create more hydrophobic bile acids (Witkowski et al., 2020; Alpuim Costa et al., 2021). The main problem of these secondary bile acids is the interaction with farmesoid X receptor (FXR) and G Protein-coupled membrane receptor 5 (TGR5), which in turn contribute to triglyceride accumulation and inflammation, leading to myocardial apoptosis, fibrosis, and subsequently cardiac damage (Calkin and Tontonoz, 2012; Huang et al., 2022).

Furthermore, TMAO, another deleterious bacterial metabolite, originated by the breakdown of some proteins like lecithin, choline and carnitine, by specific bacteria of the phyla *Firmicutes* and *Actinobacteria* (Romano et al., 2015; Huang et al., 2022). The TMAO is still not fully understood, as it can be protective or aggressive in the context of cardiovascular disease. However, the most studied mechanism of deleterious effects is the ability to interact with monocytes, causing their differentiation into macrophages and foam cells (Brown and Hazen, 2018), as well as the induction of NLRP3 inflammasome, promoting atherosclerosis and cardiac fibrosis and, therefore, increasing cardiovascular risk (Li et al., 2019; Huang et al., 2022).

Finally, SCFAs, which are carboxylic acids originating from bacterial metabolism through fermentation of fibers and non-digestible carbohydrates, produced mainly in cecum and colon (Xiong et al., 2022), are associated with numerous beneficial actions, predominantly related to anti-inflammatory characteristics. One of the main protective actions against cardiotoxicity is that some SCFAs, such as butyrate, can reduce the amount of ROS, thereby, reducing cardiac damage (Russo et al., 2019; Huang et al., 2022). This decrease of oxidative stress happens by inhibiting histone deacetylase (HDAC), which in turn, can activate Nrf2- Keap1 pathway (Nuclear erythroid-related factor 2 – Kelch-like ECH-associated protein 1). If the cell is inert, Keap 1 is blocking Nrf2, promoting ubiquitination and subsequent degradation of cells. However, if an increase in oxidative stress is detected, there is an accumulation of Nrf2 synthesis in the nucleus of the cell that, accompanied by Maf proteins, interacts with antioxidant response element (ARE), at the promoter region of antioxidative genes, originating antioxidant enzymes (González-Bosch et al., 2021). With less oxidative stress, myocardial cells apoptosis diminishes, which means a reduction in cardiac lesions.

## 5 Anti-Her2 monoclonal antibodies – trastuzumab

Di Monica M. et al. demonstrated the importance of microbiota in trastuzumab efficacy, in a mice model, as well as suggested the existence of a gut microbiota/immune mediated trastuzumab activity axis. Summing up, mice were treated with broad-spectrum antibiotics (streptomycin and vancomycin), which resulted in the diminution of *Actinobacteria*, specially *Coriobacteriaceae*, *Clostridiales*, namely *Lachnospiraceae*, *Turicibacteraceae* and *Bacteroidetes*, particularly *Prevotellaceae*, mainly SCFA-producing bacteria. Subsequently, it was noted a significant degradation of the intestinal barrier that, in turn, lead to decreased efficacy of APCs with reduced innate immune system activation. Additionally, it was observed a reduction in the concentration of IL12p70.

Afterwards, these antibiotic-treated mice were submitted to faecal material transplant (FMT) from non-antibiotic treated mice that resulted in re-establishment of intestinal microbiota homeostasis, accompanied by an augmented activation of the innate immune system, recovery of the intestinal barrier and a significant increase of IL12p70 (Di Modica et al., 2021; Di Modica et al., 2022).

Moreover, this study revealed the importance of IL12p70, a cytokine liberated by dendritic cells in response to microbiota signal, which acts through the activation of APC cells, that induce T and NK cells, originating an increase of trastuzumab efficacy (Vignali and Kuchroo, 2012).

Furthermore, the same research group evaluated the microbiota of women that do not respond to trastuzumab and, remarkably, there was an akin composition to antibiotic-treated mice, with the same phyla and taxonomic family’s changes in composition, as described above. Furthermore, this lack of responsiveness to trastuzumab was settled after a FMT from the non-responsive women to mice (Di Modica et al., 2021; Di Modica et al., 2022).

## 6 How can cardiotoxicity associated with cancer treatment be improved through the microbiota?

Cardiotoxicity is a relevant drawback both for the cancer therapy efficacy, since there is a need to postpone, adapt or interrupt the treatment regimen (Tran et al., 2020), and for the quality-of-life of cancer patients.

Therefore, it is crucial to find a way to significantly reduce cardiotoxicity. In fact, there are some measures, namely low dosages, since in some cases this cardiotoxicity is dose-dependent (e.g., DOX) (Carvalho et al., 2009), increased surveillance with regular echocardiography (Chung et al., 2013) and, even, the administration of regular drugs used in the treatment of cardiovascular pathology (Mir et al., 2023), such as statins, beta-blockers, among others.

However, these preventive measures against cardiotoxicity are still insufficient and, often compromise the well-being of disease-free patients in the future (Swain et al., 2003).

Thus, the intestinal microbiota has become an important ally in this battle against the side effects of anthracyclines and other chemotherapy drugs. Here, we propose some microbiota-based ideas that might be tested with the hope of reducing cancer treatment-induced cardiotoxicity:

1. Adjuvant therapy administration of symbiotics composed of mucins, as prebiotics, a multi-strain probiotic with *Alloprevotella*, *Rikenellaceae_RC9*, *Raoultella planticola* and *E. coli* BW25113, and a postbiotic, particularly SCFA to reduce the pro-inflammatory and oxidative microenvironment, improves the intestinal barrier integrity and increases DOX metabolism (Dubin et al., 2016; An et al., 2021; Huang et al., 2022);
2. Use of nanotechnology drug delivery to get SCFA directly into the myocardium for protection against inflammation and ROS;
3. Reproduction of the same enzyme mechanism as *Raoultella planticola* and *E. coli* BW25113 DOX metabolization (Alpuim Costa et al., 2021). Afterwards, administration of these enzymes a few hours after DOX treatment;
4. Personalized nutrition to obtain a more favorable intestinal microbiota, rich in SCFA-producing bacteria and other beneficial ones, to increase the anti-inflammatory and anti-oxidant activity (Yan et al., 2018; Huang et al., 2022);
5. Application of concurrent hyperbaric oxygen therapy with DOX treatment for increased cardioprotection (Karagoz et al., 2008).

Since there is no other manuscript or on-going clinical trial that can possibly explain the role of microbiota in the cardiotoxicity associated to trastuzumab, we hypothesize that the use of certain SCFA-producing bacteria, namely from the family’s *Coriobacteriaceae*, *Lachnospiraceae*, *Turicibacteraceae* and *Prevotellaceae* or even co-administer SCFA concomitant with trastuzumab may reduce the side effects, particularly cardiotoxicity, due to the fact that we could use lower drugs.

## 7 Conclusion

Cancer treatment is a fast-moving and dynamic field with a promising future. However, most of these therapies are still limited by their side effects, which can compromise the quality of life of cancer patients and even the quality of cancer treatment. Focusing on BC, the most used pharmacotherapy comprises anthracyclines and anti-HER-2 drugs, mainly trastuzumab, which have a well-known associated cardiotoxicity, that remains one of the main issues in BC treatment.

Recently, the microbiota is becoming more important as a potential influence on the side effects of chemotherapy on the organism. Hereby, several microbiota mechanisms that positively or negatively influence DOX-induced cardiotoxicity was described. Therefore, gut microbiota homeostasis appears to be a relevant pathway to decrease cardiotoxicity induced by cancer treatment and it relevant to support this line of research, with the goal to develop safer and more effective therapies against cancer.

## Author contributions

The present manuscript is the result of the original work by the authors. JG-N and DC: Conception and design. JG-N and IG: Writing. DC: Revision of the manuscript. All authors contributed to the article and approved the submitted version.
